# Dietary factors and biomarkers of systemic inflammation in older people: the Lothian Birth Cohort 1936

**DOI:** 10.1017/S000711451500210X

**Published:** 2015-09-07

**Authors:** Janie Corley, Janet A. M. Kyle, John M. Starr, Geraldine McNeill, Ian J. Deary

**Affiliations:** 1 Department of Psychology, University of Edinburgh, 7 George Square, EdinburghEH8 9JZ, UK; 2 Public Health Nutrition Group, Institute of Applied Health Sciences, University of Aberdeen, Polwarth Building, AberdeenAB25 2ZD, UK; 3 Centre for Cognitive Ageing and Cognitive Epidemiology, University of Edinburgh, 7 George Square, EdinburghEH8 9JZScotland, UK; 4 Royal Victoria Building, Western General Hospital, Porterfield Road, EdinburghEH4 2XU, UK

**Keywords:** Dietary patterns, Inflammation, FFQ, Cognitive ability

## Abstract

Epidemiological studies have reported inverse associations between various single healthy diet indices and lower levels of systemic inflammation, but rarely are they examined in the same sample. The aim of the present study was to investigate the potential relationships between biomarkers of systemic inflammation (C-reactive protein (CRP) and fibrinogen) and overall foods (dietary patterns), single foods (fruits and vegetables), and specific nutritive (antioxidants) and non-nutritive (flavonoids) food components in the same narrow-age cohort of older adults. The dietary intake of 792 participants aged 70 years from the Lothian Birth Cohort 1936 was assessed using a 168-item FFQ. Models were adjusted for age, sex, childhood cognitive ability, lifestyle factors and history of disease. Using logistic regression analyses, CRP (normal *v*. elevated) was favourably associated (at *P*< 0·05) with the ‘health-aware’ (low-fat) dietary pattern (unstandardised β = (0·200, OR 0·82, 95 % CI 0·68, 0·99) and fruit intake (unstandardised β = (0·100, OR 0·91, 95 % CI 0·82, 0·99), including flavonoid-rich apples (unstandardised β = (0·456, OR 0·63, 95 % CI 0·439, 0·946). Using linear regression analyses, fibrinogen (continuous) was inversely associated (at *P*< 0·05) with the Mediterranean dietary pattern (standardised β = (0·100), fruit intake (standardised β = (0·083), and combined fruit and vegetable intake (standardised β = (0·084). We observed no association between food components (antioxidant nutrients or specific flavonoid subclasses) and inflammatory markers. In the present cross-sectional study, nutrient-dense dietary patterns were associated with lower levels of systemic inflammation in older people. The results are consistent with dietary guidelines that promote a balanced diet based on a variety of plant-based foods.

Chronic low-grade inflammation has been implicated in the pathways of numerous diseases^(^ ^1^ ^,^ ^2^ ^)^. Elevated plasma levels of systemic inflammatory biomarkers such as C-reactive protein (CRP) and fibrinogen have been shown to predict CVD^(^ ^3^ ^)^, stroke, type 2 diabetes mellitus, cancer and dementia^(^ ^4^ ^,^ ^5^ ^)^. Dietary intake has long been known to play a role in the physiological response to inflammation. Therefore, nutrition may influence the development and progression of inflammatory conditions and may be useful in their prevention and treatment at a population level^(^ ^1^ ^)^. Dietary intake in relation to low-grade inflammation has been investigated in a number of ways. One approach, based on the overall diet, takes account of trends among food components, represented in dietary patterns. Findings from population studies^(^ ^6^ ^–^ ^8^ ^)^ and intervention trials^(^ ^9^ ^)^ provide evidence that a Mediterranean dietary pattern may be particularly beneficial in reducing inflammation. Recent prospective studies have confirmed that adherence to a healthy diet over time reduces the risk of long-term inflammation^(^ ^10^ ^–^ ^13^ ^)^. A second approach, which examines the role of single foods, has suggested that fruits, vegetables and whole grains are associated with lower concentrations of CRP^(^ ^6^ ^,^ ^11^ ^,^ ^14^ ^–^ ^20^ ^)^ and fibrinogen^(^ ^7^ ^,^ ^21^ ^–^ ^25^ ^)^. A third strand of research has focused on specific nutrient and non-nutrient components of foods. Dietary antioxidants such as β-carotene, Zn, Se, vitamin C and vitamin E have been shown to be associated with lower levels of disease-related markers of inflammation in adulthood^(^ ^26^ ^–^ ^30^ ^)^ and even earlier in life, in adolescence^(^ ^31^ ^)^. Non-nutritive polyphenolic compounds such as flavonoids, present in plant-based foods and drinks, particularly fruit, vegetables, tea, red wine and cocoa, have significant antioxidant and anti-inflammatory activity^(^ ^32^ ^)^, and have emerged in recent years as an important component in the relationship between diet and inflammation^(^ ^33^ ^–^ ^38^ ^)^ and with cardiovascular health^(^ ^39^ ^)^. It is unclear whether specific food components such as antioxidants and flavonoids are responsible for the apparent protective role of nutrient-dense dietary patterns and foods.

Often, any change in risk associated with high levels of inflammatory biomarkers disappears after multivariate adjustment for behavioural factors. For example, high concentrations of CRP have been reliably associated with obesity^(^ ^40^ ^–^ ^42^ ^)^, the metabolic syndrome^(^ ^43^ ^)^ and smoking^(^ ^44^ ^,^ ^45^ ^)^. In addition to behavioural correlates, there is also evidence to support a link between cognitive ability and inflammation. Not only is cognition associated with systemic inflammation in adulthood, but also poor cognitive ability earlier in life predicts increased inflammation in middle age^(^ ^46^ ^,^ ^47^ ^)^ and later life^(^ ^48^ ^)^ and an increased risk of death from inflammatory-associated diseases^(^ ^49^ ^)^. Often, full consideration is not given to the role of potential confounders, especially prior cognitive ability, as these data are rarely available.

The present study attempts to address three main gaps in the literature. First, studies that assess diet–inflammation associations using multiple dietary indicators in the same individuals are lacking. Without this, misleading conclusions can be drawn about the putatively protective role of a single dietary measure. Second, there are few studies conducted exclusively within old age groups. Ageing is associated with increases in several inflammatory markers, and there is strong evidence that these markers influence age-associated pathology^(^ ^50^ ^)^. Third, we examined diet–inflammation associations in a well-characterised community-dwelling Scottish cohort, of mean age 70 years, for whom there were validated intelligent quotient (IQ) scores from youth, in order to control for prior cognitive ability. We examined two commonly used circulating markers of inflammation (plasma CRP and fibrinogen) that measure different aspects of the inflammatory process, in the same elderly sample, with a view to determining whether the inverse diet–inflammation relationships previously reported are a result of dietary patterns, single foods, or more specific aspects of nutrition.

## Methods

### Study population

The Lothian Birth Cohort 1936 (LBC1936) study includes 1091 men and women, all of whom were born in 1936 and who were living independently in the community at about 70 years of age. Almost all participants were resident in Edinburgh and the surrounding Lothian region at recruitment in older age. Early-life (mean age 11 years) intelligence test data were available for most of this sample, because most were surviving participants of the Scottish Mental Survey of 1947^(^ ^51^ ^)^. Assessment in later life took place between 2004 and 2007 when participants were aged about 70 years (mean 69·5 (sd 0·8)). Full recruitment and testing procedures have been reported in an open-access protocol paper^(^ ^52^ ^,^ ^53^ ^)^. The assessment at age 70 years involved detailed cognitive, biomedical and psychosocial testing. FFQ data were available for 882 participants. We excluded ninety participants with a CRP measure >10 mg/l (to omit possible acute illness). After exclusion, 792 individuals remained for the present analyses. The study was conducted according to the guidelines laid down in the Declaration of Helsinki, and all procedures involving human subjects/patients were approved by the Multi-Centre Research Ethics Committee for Scotland (MREC/01/0/56) and from the Lothian Research Ethics Committee for Scotland (LREC/2003/2/29). Written informed consent was obtained from all subjects/patients.

### Assessment of dietary intake

Dietary data were derived from the Scottish Collaborative Group 168-item FFQ, version 7.0^(^ ^54^ ^,^ ^55^ ^)^. This semi-quantitative FFQ was developed for use in older adults (see http://www.foodfrequency.org.uk)^(^ ^56^ ^)^. A common unit or portion size for each food or drink item is specified, and responses are marked according to a nine-point scale, ranging from ‘rarely or never’ to ‘7+ per d’ to describe the typical amount and frequency of each food consumed. All participants were asked to complete the FFQ at home and return it by post. Estimation of food and nutrient intake was carried out at the University of Aberdeen using an in-house programme and the latest available information in the UK food composition tables. The repeatability and validity of the FFQ was demonstrated against 4 d weighed food diaries; dietary intake in later life was found to be reasonably stable in the short term, and the authors reported good validity for most nutrients in community-dwelling older populations^(^ ^54^ ^,^ ^57^ ^)^. Intake of antioxidant nutrients and flavonoids, and dietary pattern scores were adjusted for overall energy intake using the residual method^(^ ^58^ ^)^.

#### Dietary patterns

Dietary patterns were extracted via principal components analysis with orthogonal rotation from all FFQ items. Further description of their ascertainment can be found in Mõttus *et al.* ^(^ ^59^ ^)^. The Mediterranean dietary pattern was primarily defined by greater consumption of vegetables (such as leeks or courgettes, broccoli, and salad vegetables), fish, poultry, pasta, rice, water, tomato-based sauces, oil and vinegar dressing, and beans. The ‘health-aware’ dietary pattern was mainly characterised by a high intake of fruits (e.g. apples, bananas, tinned fruits, oranges and others) and carrots, and low consumption of meat products (bacon or gammon, pork or lamb, and sausages), eggs, and spirits or liqueurs. Participants obtained a score for each dietary pattern, indicating the degree to which the individual's diet conformed to that pattern, where the mean score is 0.

#### Fruit and vegetables

Total (fresh) fruit and vegetable intakes were calculated independently and in combination. Fruit included fresh fruit salad, apples, bananas, oranges, satsumas, grapefruit, pears, peaches, nectarines, kiwi fruit, grapes, strawberries, melons and other fresh fruits. Vegetables included peas, green beans, carrots, cabbage, Brussels sprouts, spinach, spring greens, leeks, courgettes, cauliflower, swede, turnips, sweetcorn, onions, tomatoes, sweet peppers, and other salad vegetables such as lettuce, cucumber, etc.

#### Flavonoid-rich foods

Intakes of flavonoid-rich foods (chocolate, apples and citrus fruits) and drinks (tea and red wine) were analysed.

#### Flavonoid subclasses

Intakes of a selection of five flavonoid subclasses (flavonols, flavones, catechins, proanthocyanidin type B and flavanones) were estimated using a UK flavonoid database, which included 396 items^(^ ^60^ ^)^.

#### Antioxidant nutrients

Dietary intake of antioxidant nutrients (vitamin C, vitamin E, β-carotene, Zn and Se) was estimated using the FFQ data.

### Assessment of inflammatory markers

Serum CRP (mg/l) and fibrinogen (g/l) were extracted from blood samples that were collected intravenously by nurses as part of the participants' clinical assessment. The CRP assay was performed using a dry-slide immuno-rate method on OrthoFusion 5.1 F.S analysers (Ortho Clinical Diagnostics). The CRP assay method has low sensitivity in the lower range of CRP values; approximately 58 % of the participants fell into a single lowest category (1·5 mg/l). Therefore, in the analyses, and as in Mõttus *et al.* ^(^ ^59^ ^)^, all CRP values were collapsed into two categories: ≤ 3 mg/l (normal; including the measured values of 1·5 and 3 mg/l) and >3 mg/l (elevated; including the rest of the values). Besides being meaningful for the current distribution of CRP values, the relevance of the 3 mg/l cut-off has been suggested for the prediction of CVD^(^ ^61^ ^)^. The fibrinogen assay was performed using an automated Clauss assay (TOPS coagulometer; Instrumentation Laboratory). The range of fibrinogen values was 1·5–6·0 g/l.

### Assessment of covariates

General demographic information, including sex (1 = male, 2 = female), age (exact age in days at the time of assessment) and years of full-time education, was assessed and coded accordingly. Childhood cognitive ability was derived from scores on the Moray House Test taken at age 11 years^(^ ^52^ ^)^. The Moray House Test is a group-administered test of general intelligence. This test was concurrently validated against the Terman–Merrill revision of the Binet scales^(^ ^62^ ^)^. The scores were converted to standard IQ-type scores for the whole sample (*n* 1091), with a mean value of 100 and a standard deviation of 15. Adult occupational social class was derived from each participant's highest reported occupation and classified into one of six categories ranging from I (professional occupations) to V (unskilled occupations), with III divided into IIIN (non-manual) and IIIM (manual)^(^ ^63^ ^)^. For data analysis, occupational social classes IV and V were combined due to the small number of participants in each class. Height and weight were measured by trained research nurses as part of a physical examination. BMI was calculated as weight (kg) divided by height squared (m^2^). Smoking status was coded as 0 (never smoker), 1 (ex-smoker) or 2 (current smoker). Physical activity was the number of days of sport or physical exercise (e.g. dancing or brisk walking) per average month. A self-reported history of CVD, hypertension, stroke and diabetes was recorded by a trained interviewer at the time of assessment and coded as dichotomous variables (0 = no, 1 = yes). The cholesterol:HDL-cholesterol ratio (Chol:HDL ratio) was obtained from a blood sample drawn on the day of assessment. The Chol:HDL ratio provides a strong prediction of CHD risk; a lower ratio is clinically more desirable.

### Statistical analyses

All statistical analyses were performed using SPSS version 19 (IBM). The associations between potential covariates and inflammatory biomarkers were tested using regression analyses. For CRP (elevated *v*. normal), we performed logistic regression analyses, and for fibrinogen (continuous), we performed linear regression analyses. In the main analyses, we used three models to examine the associations between dietary predictor variables and inflammatory biomarkers. All categorical variables were treatment coded. Model 1 included sex and exact age in days at the time of assessment. In model 2, IQ at age 11 years (age 11 IQ) was added to adjust for any confounding effects of childhood cognitive ability; childhood IQ was previously found to be an independent predictor of CRP in this sample^(^ ^48^ ^)^. In model 3, to control for potentially confounding lifestyle factors and health variables, we added occupational social class, BMI, physical activity, smoking status, history of CVD, hypertension, Chol:HDL ratio, stroke and diabetes to model 2. The effects are reported using unstandardised β regression coefficients for CRP analyses, and standardised β regression coefficients for fibrinogen analyses. *P* values are reported. The 0·05 level of significance was used for all data analyses.

## Results

The characteristics of the study participants are presented in Table 1. A total of 792 participants (48 % men) with a mean age of 69·5 years (sd 0·85) were included in the analyses. Nearly half (48 %) were never smokers and 9 % were current smokers, the mean BMI was 27·4, and 23·6 % reported a history of CVD, 38·3 % of hypertension, 3·5 % of stroke and 7·2 % of diabetes. Of the participants, 42 % had an elevated CRP level of ≥ 4 mg/l. Pearson's correlation coefficient (*r*) between inflammatory biomarkers, CRP and fibrinogen was 0·287 (*P*< 0·001). Table 2 presents the unadjusted associations (derived from logistic/linear regression analyses) between CRP, fibrinogen and covariates. A higher level of CRP was associated with being older (even in this narrow-age cohort), a lower age 11 IQ, a history of smoking, a higher BMI (all *P*< 0·01), a less-professional social class (*P*= 0·028), a diagnosis of hypertension (*P*< 0·001) and diabetes (*P*= 0·02), and a higher (less-desirable) Chol:HDL ratio (*P*= 0·004), but not with CVD or stroke. A higher level of fibrinogen was associated with being younger (*P*= 0·002), a lower age 11 IQ (*P*= 0·018), a history of smoking (*P*= 0·005), a higher BMI (*P*= 0·047), and a history of hypertension (*P*= 0·002), stroke (*P*= 0·002) and diabetes (*P*= 0·001), but not with CVD or Chol:HDL ratio. Table 3 presents the unadjusted associations (derived from linear regression analyses) between the dietary patterns and covariates. A higher Mediterranean pattern score was associated with being younger (*P*= 0·015), a higher age 11 IQ (*P*= 0·002), less smoking (*P*< 0·001), being female (*P*= 0·002) and a lower Chol:HDL ratio (*P*= 0·028). A higher health-aware dietary pattern score was associated with less smoking (*P*< 0·001), a lower BMI (*P*= 0·015), more physical activity (*P*= 0·037) and being female (*P*< 0·001).

**Table 1:** Characteristics of the study population from the Lothian Birth Cohort 1936 (LBC1936; *n* 792) (Number of participants; mean values and standard deviations; number of participants and percentages)

Characteristics	*n*	Mean	sd
Demographic and lifestyle			
Age (years)	792	69·5	0·85
Male	792		
*n*	383		
%	48·4		
Age 11 IQ	748	101·6	14·0
Education (full time, years)	792	10·8	1·1
Occupational class	776		
I			
*n*	147		
%	18·9		
II			
*n*	303		
%	39·0		
IIIN			
*n*	187		
%	24·1		
IIIM			
*n*	116		
%	14·9		
IV and V*			
*n*	23		
%	3·0		
Smoking status	792		
Never smoker			
*n*	383		
%	48·4		
Ex-smoker			
*n*	337		
%	42·6		
Current smoker			
*n*	72		
%	9·1		
BMI	791	27·4	4·1
Physical activity (d/month)	779	7·88	8·1
CVD diagnosis	792		
*n*	187		
%	23·6		
Hypertension diagnosis	792		
*n*	303		
%	38·3		
Chol:HDL ratio	685		
< 3·5			
*n*	287		
%	41·9		
3·5– < 5			
*n*	325		
%	47·4		
≥ 5			
*n*	73		
%	10·7		
Stroke diagnosis	792		
*n*	28		
%	3·5		
Diabetes diagnosis	792		
*n*	57		
%	7·2		
Plasma markers of inflammation			
CRP†	766		
Normal ( ≤ 3 mg/l)			
*n*	448		
%	58·5		
Elevated (>3 mg/l)			
*n*	318		
%	41·5		
Fibrinogen (g/l)	770	3·2	0·58
Dietary intake			
Dietary patterns (factor scores)‡			
Mediterranean dietary pattern	792	0·01	0·99
Health-aware dietary pattern	792	0·03	1·0
Fruit and vegetables (measures per d)			
Total fruit	792	2·5	2·4
Total vegetables	792	3·1	2·4
Total fruit and vegetables	792	5·6	4·0
Flavonoid-rich foods (measures per d)			
Tea	790	2·5	2·0
Red wine	791	0·33	0·76
Chocolate	790	0·55	0·70
Apples	792	0·44	0·59
Citrus fruits	792	0·41	0·58
Dietary flavonoids‡			
Flavonols	792	37·6	24·5
Flavones	792	0·29	0·20
Catechins	792	185·5	137·9
Proanthocyanidins	792	52·6	34·3
Flavonones	792	25·6	25·7
Dietary intakes (per d)‡			
Vitamin C (mg)	792	107·3	60·3
Vitamin E (μg)	792	8·0	3·6
β-Carotene (μg)	791	2778·0	2112·3
Zn (mg)	792	8·8	2·8
Se (μg)	792	48·2	19·2

Age 11 IQ, intelligent quotient at age 11 years; IIIN, non-manual; IIIM, manual; Chol:HDL ratio, cholesterol:HDL-cholesterol ratio; CRP, C-reactive protein.

* Occupational social classes IV and V were combined due to the small number of participants in each class.

† Participants with a CRP measure >10 mg/l were excluded (*n* 90) to omit possible acute illness.

‡ Adjusted for total energy intake.

**Table 2:** Univariate associations between C-reactive protein (CRP) and fibrinogen levels and covariates (Unstandardised and standardised β regression coefficients; mean values and standard deviations)

	CRP (mg/l)*	Fibrinogen (g/l)†
Characteristics	Unstandardised β	Mean	sd	*P*	Standardised β	Mean	sd	*P*
Age	0·215			< 0·001	− 0·112			0·002
Age 11 IQ	− 0·128			0·001	− 0·088			0·018
BMI	0·262			< 0·001	0·072			0·047
Physical activity‡	− 0·026			0·339	− 0·049			0·180
Sex	0·009			0·795	0·057			0·115
Male		3·38	2·4			3·17	0·59	
Female		3·45	2·5			3·24	0·56	
Occupational class	0·080			0·028	0·044			0·223
I		3·12	2·2			3·15	0·57	
II		3·38	2·5			3·20	0·58	
IIIN		3·38	2·4			3·24	0·56	
IIIM		4·04	2·5			3·18	0·63	
IV and V§		3·41	2·4			3·36	0·48	
Smoking status	0·088			0·015	0·103			0·004
Never smoker		3·17	2·2			3·18	0·56	
Ex-smoker		3·51	2·5			3·18	0·57	
Current smoker		4·32	2·9			3·48	0·60	
CVD	0·026			0·473	0·050			0·165
No		3·40	2·4			3·19	0·56	
Yes		3·48	2·4			3·26	0·63	
Hypertension	0·129			< 0·001	0·112			0·002
No		3·21	2·4			3·15	0·54	
Yes		3·77	2·4			3·29	0·62	
Chol:HDL ratio	0·109			0·004	0·045			0·244
< 3·5		3·27	2·4			3·16	0·57	
3·5– < 5·0		3·46	2·4			3·23	0·59	
≥ 5·0		4·32	2·7			3·20	0·57	
Stroke	0·003			0·934	0·112			0·002
No		3·40	2·4			3·19	0·56	
Yes		3·96	3·1			3·54	0·81	
Diabetes	0·084			0·020	0·117			0·001
No		3·35	2·4			3·19	0·57	
Yes		4·33	2·7			3·45	0·63	

Age 11 IQ, intelligent quotient at age 11 years; IIIN, non-manual; IIIM, manual; Chol:HDL ratio, cholesterol:HDL-cholesterol ratio.

* Unstandardised β regression coefficients and *P* values were derived from logistic regression analyses (CRP: normal ( ≤ 3 mg/l) *v*. elevated (>3 mg/l)).

† Standardised β regression coefficients and *P* values were derived from linear regression analyses.

‡ Physical activity was the number of days of sport or physical exercise per month.

§ Occupational social classes IV and V were combined due to the small number of participants in each class.

**Table 3:** Univariate associations between the Mediterranean and health-aware dietary pattern factor scores and covariates (Unstandardised and standardised β regression coefficients; mean values and standard deviations)

	Mediterranean	Health-aware
Characteristics	Unstandardised β	Mean	sd	*P*	Standardised β	Mean	sd	*P*
Age	− 0·087			0·015	0·038			0·291
Age 11 IQ	0·112			0·002	− 0·039			0·290
BMI	− 0·032			0·370	− 0·087			0·015
Physical activity*	0·053			0·142	0·075			0·037
Sex	0·112			0·002	0·331			< 0·001
Male		− 0·10	0·86			− 0·31	1·0	
Female		0·12	1·1			0·35	0·91	
Occupational class	− 0·272			< 0·001	− 0·002			0·945
I		0·44	1·1			− 0·07	1·1	
II		0·12	1·1			0·08	0·95	
IIIN		− 0·17	0·78			0·20	0·90	
IIIM		− 0·42	0·71			− 0·23	0·97	
IV and V†		− 0·25	0·96			− 0·04	1·6	
Smoking status	− 0·070			0·048	− 0·191			< 0·001
Never smoker		0·03	0·91			0·19	0·94	
Ex-smoker		0·06	1·1			− 0·03	1·0	
Current smoker		− 0·35	0·89			− 0·52	1·1	
CVD	− 0·030			0·392	− 0·026			0·466
No		0·03	1·0			0·05	0·99	
Yes		− 0·04	0·85			− 0·02	1·0	
Hypertension	− 0·038			0·283	0·026			0·461
No		0·042	1·1			0·01	0·97	
Yes		− 0·036	0·85			0·064	1·1	
Chol:HDL ratio	− 0·084			0·028	0·000			0·996
< 3·5		0·99	1·1			0·03	1·0	
3·5– < 5·0		− 0·033	0·90			0·04	0·98	
≥ 5·0		− 0·15	0·80			0·02	1·2	
Stroke	− 0·028			0·438	0·015			0·681
No		0·01	0·99			0·03	1·0	
Yes		− 0·13	0·86			0·11	1·1	
Diabetes	− 0·053			0·134	0·035			0·320
No		0·02	0·98			0·02	1·0	
Yes		− 0·18	0·99			0·16	0·95	

Age 11 IQ, intelligent quotient at age 11 years; IIIN, non-manual; IIIM, manual; Chol:HDL ratio, cholesterol:HDL-cholesterol ratio.

* Physical activity was the number of days of sport or physical exercise per month.

† Occupational social classes IV and V were combined due to the small number of participants in each class.

Table 4 shows the results of the main regression analyses for all dietary measures (variously adjusted in three models).

**Table 4:** Multivariate associations between C-reactive protein (CRP) and fibrinogen levels and dietary factors at age 70 years (Unstandardised and standardised β regression coefficients)

	CRP (mg/l)*	Fibrinogen (g/l)†
	Model 1‡	Model 2§	Model 3∥	Model 1‡	Model 2§	Model 3∥
Predictors	Unstandardised β	Unstandardised β	Unstandardised β	Standardised β	Standardised β	Standardised β
Dietary patterns						
Mediterranean	− 0·141	− 0·120	− 0·078	− 0·133***	− 0·123**	− 0·100*
Health-aware	− 0·291**	− 0·293**	− 0·200*	0·023	0·014	0·009
Fruit and vegetables						
Total fruit	− 0·109**	− 0·117**	− 0·100*	− 0·088*	− 0·089*	− 0·083*
Total vegetables	− 0·065	− 0·060	− 0·033	− 0·072*	− 0·071	− 0·057
Total FV	− 0·062**	− 0·062**	− 0·044	− 0·097**	− 0·096*	− 0·084*
Flavonoid-rich foods						
Tea	0·015	− 0·005	− 0·010	− 0·007	− 0·015	− 0·014
Red wine	− 0·010	− 0·032	0·048	− 0·133***	− 0·101**	− 0·073
Chocolate	− 0·067	− 0·016	− 0·048	− 0·078*	− 0·068	− 0·059
Apples	− 0·441**	− 0·522**	− 0·456*	− 0·007	− 0·036	− 0·037
Citrus fruits	− 0·369*	− 0·395*	− 0·337	− 0·055	− 0·032	− 0·018
Flavonoids						
Flavonols	0·000	− 0·002	− 0·002	− 0·003	− 0·015	0·010
Flavones	0·194	0·088	− 0·190	− 0·025	− 0·048	− 0·065
Catechins	0·000	0·000	0·000	0·000	− 0·007	0·019
Proanthocyanidins	0·000	− 0·002	− 0·002	− 0·029	− 0·032	− 0·004
Flavanones	− 0·008*	− 0·010*	− 0·007	− 0·049	− 0·028	− 0·013
Antioxidant nutrients						
Vitamin C	− 0·003*	− 0·003*	− 0·002	− 0·099**	− 0·093*	− 0·074
Vitamin E	0·005	0·007	0·028	− 0·010	− 0·005	0·007
β-Carotene	0·000	0·000	0·000	− 0·042	− 0·048	− 0·054
Zn	0·000	0·000	− 0·017	− 0·055	− 0·056	− 0·054
Se	− 0·004	− 0·004	− 0·005	− 0·051	− 0·051	− 0·055

FV, fruit and vegetables; age 11 IQ, intelligent quotient at age 11 years.

*P< 0·05, **P< 0·01, ***P< 0·001.

* Unstandardised β regression coefficients and *P* values were derived from logistic regression analyses (CRP: normal ( ≤ 3 mg/l) *v*. elevated (>3 mg/l)).

† Standardised β regression coefficients and *P* values were derived from linear regression analyses.

‡ Model 1 was adjusted for age and sex.

§ Model 2 was adjusted for age, sex and age 11 IQ.

∥ Model 3 was adjusted for age, sex, age 11 IQ, occupational social class, BMI, physical activity, cholesterol:HDL-cholesterol ratio, smoking status, and history of CVD, hypertension, stroke and diabetes.

### Diet and C-reactive protein

First, we performed logistic regression analyses on the associations between diet and CRP (normal *v*. elevated levels). In the basic age- (in d) and sex-adjusted models (model 1), a lower CRP level ( ≤ 3 mg/l) was associated with a higher health-aware dietary pattern score, a higher intake of fresh fruit including flavonoid-rich apples and citrus fruits, and combined fruit and vegetables, dietary vitamin C, and flavanones (found in citrus fruits). Additionally adjusting for the potential confounding effects of higher childhood cognitive ability (model 2) made little difference to effect sizes. Further adjustment for occupational social class, BMI, physical activity, smoking status, Chol:HDL ratio, and history of CVD, stroke and diabetes (model 3) caused some of these associations to lose significance. In the final model, the dietary measures associated with a lower CRP concentration (at *P*< 0·05) were the ‘health-aware’ (low-fat) dietary pattern (unstandardised β = (0·200, OR 0·82, 95 % CI 0·68, 0·99) and fruit intake (unstandardised β = (0·100, OR 0·91, 95 % CI 0·82, 0·99), including flavonoid-rich apples (unstandardised β = (0·456, OR 0·63, 95 % CI 0·439, 0·946). No significant association was found with vegetable intake (independently) and CRP.

### Diet and fibrinogen

Second, we performed linear regression analyses on the associations between diet and fibrinogen. In the basic age- (in d) and sex-adjusted models (model 1), a lower fibrinogen level was associated with a higher Mediterranean dietary pattern score and a higher intake of fruit and vegetables, flavonoid-rich red wine and chocolate, and diet-derived vitamin C. With the exception of a higher intake of vegetables and chocolate, these inverse associations remained significant after controlling for childhood cognitive ability (in model 2). Further adjustment for lifestyle and health covariates (in model 3) caused some associations to lose significance. Robust inverse associations (all at *P*< 0·05) were observed between fibrinogen and the Mediterranean dietary pattern (standardised β = (0·100), fruit intake (standardised β = (0·083), and combined fruit and vegetable intake (standardised β = (0·084).

## Discussion

In the present large sample of community-dwelling older adults aged approximately 70 years, healthy dietary patterns rich in fresh produce, especially fruit, were associated with lower concentrations of two common biomarkers of systemic low-grade inflammation. Closer adherence to a Mediterranean diet was related to lower fibrinogen, but not CRP concentrations. Instead, a lower CRP level was associated with a ‘prudent’ (health-aware) dietary pattern comprising fruit and low-fat foods. It is noteworthy that these relationships remained significant after adjusting for variables that reflected a healthy lifestyle, such as smoking, BMI and physical activity, factors strongly associated with a high concentration of inflammatory biomarkers^(^ ^41^ ^,^ ^44^ ^)^, and for a history of major chronic diseases. Interestingly, consumption of vegetables *per se* and specific food components (antioxidant nutrients and various subclasses of flavonoids) failed to show statistically significant relationships with either of the biomarkers. A key finding from the present study, and contrary to expectations, was that the significant relationship between diet and inflammation remained after adjusting for childhood cognitive ability, previously shown to be an important predictor of both diet choices^(^ ^64^ ^,^ ^65^ ^)^, including flavonoid consumption^(^ ^66^ ^)^, and inflammation^(^ ^46^ ^–^ ^48^ ^)^ in adulthood, and inversely associated with both CRP and fibrinogen in this sample. The data suggest that habitual dietary patterns may independently relate to inflammation in later life.

A large literature links healthy dietary patterns, especially the Mediterranean diet, with positive health outcomes^(^ ^67^ ^)^, including lower levels of systemic inflammation. A recent systematic review by Barbaresko *et al.* ^(^ ^4^ ^)^ has concluded that fruit and vegetable-based ‘healthy’ dietary patterns are associated with lower biomarkers of inflammation including CRP, and that this finding is particularly well-supported by intervention studies with the Mediterranean diet^(^ ^68^ ^–^ ^70^ ^)^. In a 2-year intervention study, subjects who were administered a Mediterranean diet (rich in vegetables, fruit, nuts, olive oil and whole grains) experienced a significant decline in CRP levels, which was not found in the control group^(^ ^68^ ^)^. This applied to all biomarkers of inflammation measured, particularly CRP. Closer adherence to a Mediterranean-type diet in observational studies has been shown to be associated with lower levels of CRP and fibrinogen^(^ ^6^ ^,^ ^7^ ^,^ ^21^ ^,^ ^24^ ^,^ ^71^ ^)^, thus providing more evidence that this dietary pattern and its constituents may help to lower low-grade inflammation. One further study found that CRP levels, although not significantly associated with the Spanish Mediterranean diet, were lowest in subjects with the highest consumption of olive oil and nuts, the major components of this pattern^(^ ^72^ ^)^. However, some trials in Northern European populations, such as Germany, have found no effect of the Mediterranean diet on the levels of CRP or fibrinogen^(^ ^73^ ^)^. It is possible that the observed associations between ‘prudent’ dietary patterns and inflammatory markers may be indirect. Healthy foods and snacks may be consumed at the expense of unhealthy, sugary or fatty (pro-inflammatory) foods. Following intake of energy-dense, nutrient-poor, processed foods, meal-induced inflammation has been evidenced by immediate increases in inflammatory biomarkers such as CRP^(^ ^74^ ^)^. In population studies, Western-type dietary pattern components such as red meat, high-fat dairy and other sources of saturated fat, and refined carbohydrates show positive associations with biomarkers such as CRP and fibrinogen^(^ ^2^ ^,^ ^18^ ^,^ ^20^ ^,^ ^22^ ^,^ ^75^ ^,^ ^76^ ^)^.

Studying dietary patterns has a number of advantages over the ‘single food or nutrient’ approach. Foods and nutrients are rarely eaten in isolation, whereas dietary patterns capture the complexity of diets and synergistic interactions among nutrients in addition to different food sources of the same nutrient^(^ ^77^ ^)^. However, a large number of epidemiological and intervention studies have focused specifically on fruit and vegetable intake as single food components. Fruit and vegetables are a rich source of beneficial compounds including vitamins, carotenoids, polyphenols and other bioactive compounds, which make them a food group with a high dietary antioxidant capacity and multiple anti-inflammatory actions^(^ ^78^ ^,^ ^79^ ^)^. No association was found between CRP and fruit and vegetables intake in a sample at high risk for CVD^(^ ^72^ ^)^; however, many other observational studies have reported potentially beneficial effects of fruit and vegetable intake on CRP in adulthood^(^ ^15^ ^–^ ^17^ ^,^ ^80^ ^)^ even after adjusting for BMI, smoking and other covariates. CRP has been shown to decrease as fruit and vegetables consumption increases. One intervention study placed healthy non-smoking men on diets containing only two servings per d of fruits and vegetables for 4 weeks and then placed them on diets of increasing fruit and vegetable consumption for another 4 weeks. Those who were randomised to eight fruit/vegetable servings (but not five servings) per d had a significant decline in CRP levels^(^ ^81^ ^)^. The lack of association of vegetable intake with the biomarkers of inflammation in our sample may be due to cultural differences; in Scotland, vegetable consumption is lower than in the UK average^(^ ^82^ ^)^ and markedly lower than in Mediterranean populations where, on average, greater importance is placed on fresh produce^(^ ^83^ ^)^. Our data support the findings of two other UK studies: the British Regional Heart Study, which reported inverse associations with CRP and fruit intake but not with vegetable intake in older men^(^ ^25^ ^)^, and a longitudinal (6-year) investigation finding no independent association with overall vegetable intake^(^ ^10^ ^)^. Deep-rooted cultural differences in diet can make comparisons between studies problematic. Furthermore, many studies reporting significant associations with biomarkers have assessed fruit and vegetable intake in combination only^(^ ^14^ ^,^ ^80^ ^,^ ^84^ ^)^; therefore, it is unclear whether a particular dietary component is driving the associations.

Anti-inflammatory benefits with increased fruit and vegetable intake have often been attributed to the effect of specific antioxidant nutrients, such as β-carotene, Zn and vitamin C, since oxidative stress is an underlying mechanism for several chronic diseases^(^ ^85^ ^,^ ^86^ ^)^. Brighenti *et al.* ^(^ ^78^ ^)^ reported that CRP levels were progressively lower with increasing levels of antioxidant capacity of the diet, estimated from dietary intake of fruits, vegetables and other foods. However, a Catalonian study has failed to find any association between CRP and β-carotene, vitamin C or vitamin E^(^ ^87^ ^)^. Among the five antioxidant nutrients assessed in the present analysis, only one (vitamin C) was found to have a potentially beneficial effect on inflammation (fibrinogen). This finding was probably due to the vitamin C content in fruit; however, this association narrowly missed significance following multivariate adjustment. In another study, reduced CRP and fibrinogen concentrations were associated with a higher intake of dietary vitamin C, but not with other antioxidant nutrients tested^(^ ^88^ ^)^. A decrease in inflammation status associated with a higher intake of dietary vitamin C is supported by other population studies in middle-aged and elderly people^(^ ^16^ ^,^ ^25^ ^)^and in some clinical trials^(^ ^29^ ^)^. In general, the results of the present study suggest that, with the possible exception of food-derived vitamin C, intake of antioxidant nutrients does not explain the inverse diet–inflammation associations. Furthermore, epidemiological studies have consistently shown that increased fruit and vegetable intakes are associated with a lower risk of cardiovascular events^(^ ^89^ ^,^ ^90^ ^)^. Yet, supplementation with several of these antioxidant nutrients in clinical trials and intervention studies has not demonstrated a concomitant decline in the risk of CVD^(^ ^91^ ^,^ ^92^ ^)^.

Previous studies have found that intake of dietary flavonoids is inversely associated with inflammatory diseases^(^ ^32^ ^)^ and serum CRP concentrations^(^ ^34^ ^,^ ^42^ ^)^. Of the flavonoid-rich foods and drinks assessed in this sample, only apples were associated with lower inflammation (CRP). Consistent with the results of the present investigation, Chun *et al.* ^(^ ^34^ ^)^ observed that consumption of apples, a rich source of flavonoids, was associated with lower CRP concentrations. Several clinical trials have supported the link between flavonoid consumption and reduced CRP concentrations^(^ ^93^ ^)^, but not all^(^ ^94^ ^)^. We observed no relationship between several flavonoid subclasses, contrary to other studies. For example, several flavonoid subclasses (flavones, flavanones and total flavonoids) were associated with lower concentrations of some inflammatory biomarkers in the Nurses' Health Study^(^ ^38^ ^)^. However, data on the associations between flavonoids and inflammation are still considered controversial, and inaccurate estimates of flavonoid intake from the diet are based on often incomplete flavonoid food composition data and, therefore, inconsistent findings may arise^(^ ^34^ ^)^.

The present study was limited by the cross-sectional nature of the data; therefore, the observed associations might not be causal. Given the older age of participants, reverse causation is possible. Certain low-grade inflammatory states such as mild malaise could have an easy impact on diet, and some individuals with a history of chronic disease (related to inflammation, perhaps) might have altered their diets according to health recommendations before assessment. Other limitations in using this dataset include the use of a self-report measure of dietary consumption; self-reports may be biased. However, the validity of the FFQ has previously been reported in an old-age sample^(^ ^54^ ^,^ ^55^ ^)^. As a self-selected volunteer sample of a narrow older age and specific geographical location with high cultural homogeneity, the findings from the LBC1936 cohort may not generalise to other populations.

A strength of the present study was the use of a well-characterised sample population, with available measures of diet, biomarkers of interest, and comprehensive health and lifestyle information. We used an age-homogeneous population, minimising age and cohort effects. This is important as CRP and fibrinogen increase with age, and the effect of potential confounding factors can vary according to lifelong exposure^(^ ^5^ ^)^. Some chronic low-grade inflammation patterns found in the elderly may be related to co-morbidities; however, it is also observed in ‘successful ageing’^(^ ^50^ ^)^. The heterogeneity among reported associations with diet in studies may be due to the use of different inflammatory markers and to the different physiological mechanisms involving each biomarker^(^ ^4^ ^)^. At present, there is no consensus as to which markers of inflammation best represent low-grade inflammation^(^ ^5^ ^)^. The present data expand previous knowledge obtained from observational studies, and lend further support to the evidence suggesting that diet may play a role in mediating the body's biological response to inflammation. However, further longitudinal and intervention studies are needed in order to determine a causal link. Because fruit and vegetable intake is consistently associated with a decreased risk of chronic diseases, public health strategies to improve fruit and vegetable intake should be encouraged. Dietary changes remain a low-risk intervention strategy.

In conclusion, our findings suggest that a Mediterranean dietary pattern and a ‘prudent’ (health-aware) dietary pattern, rich in fresh produce, are associated with lower plasma concentrations of CRP and fibrinogen in older age. The data are not supportive of a beneficial, independent effect of antioxidant nutrients or flavonoids. Dietary patterns represent a broader picture of food and nutrient consumption, and thus may be more predictive of disease risk than individual foods or nutrients.
